# Complement profiling for treatment outcomes in pulmonary TB

**DOI:** 10.3389/fimmu.2026.1679947

**Published:** 2026-02-11

**Authors:** Nathella Pavan Kumar, Arul Nancy P., Syed Hissar, Shanmugam Sivakumar, Vijay Viswanathan, Ramalingam Bethunaickan, Hardy Kornfeld, Subash Babu

**Affiliations:** 1 Indian Council of Medical Research (ICMR)- National Institute for Research in Tuberculosis, Chennai, India; 2 Academy of Scientific and Innovative Research, Ghaziabad, India; 3 National Institutes of Health-NIRT- International Center for Excellence in Research, Chennai, India; 4 Prof. M. Viswanathan Diabetes Research Center, Chennai, India; 5 University of Massachusetts Chan Medical School, Worcester, MA, United States; 6 Laboratory of Parasitic Diseases (LPD), of National Institute of Allergy and Infectious Diseases (NIAID), National Institutes of Health (NIH), Bethesda, MD, United States

**Keywords:** tuberculosis, compliment proteins, unfavorable TB treatment outcomes, prognostic markers, multiplex ELISA

## Abstract

**Introduction:**

The complement system plays a vital role in the immune response against tuberculosis (TB), aiding in the recognition and clearance of Mycobacterium tuberculosis. However, its imbalance can result in either insufficient immune activation or excessive inflammation, both of which may contribute to poor treatment outcomes.

**Methods:**

This study investigates whether baseline complement profiles are associated with unfavorable treatment responses in pulmonary TB patients. Using the Magpix multiplex cytokine assay, plasma levels of complement components (C2, C3, C3b/iC3b, C4, C4b, C5, C5a, C1q, MBL) and regulatory proteins (Factor B, Factor D, Factor H, Factor I) were measured in TB patients with poor treatment outcomes (n=68) and disease-free controls (n=108).

**Results:**

At both baseline (pre-treatment) and month two of anti-TB therapy, cases had significantly elevated levels of C3, C3b, C4b, C5, C5a, and C1q, and reduced levels of Factor B and Factor H compared to controls. Regression modelling revealed that C3, C3b, C4b, C5, C5a and C1q were associated with increased risk of unfavorable outcomes in unadjusted and adjusted analyses in the study cohort.

**Discussion:**

These findings suggest that early and sustained complement activation, particularly through the classical pathway, is associated with adverse outcomes in TB. Complement dysregulation may thus serve as a potential prognostic marker for identifying individuals at risk of poor treatment response.

## Introduction

Tuberculosis (TB), caused by *Mycobacterium tuberculosis* (*Mtb*), remains a significant global health challenge and one of the leading infectious causes of mortality worldwide, despite ongoing control efforts. The pathogenesis of TB is shaped by intricate interactions between the host immune system and *Mtb*, which can lead to either latent infection or active disease (1). Effective immune control of *Mtb* requires a delicate balance: while the immune response aims to eliminate the pathogen, *Mtb* employs various strategies to evade detection and persist within the host (2).

The complement system, a crucial component of innate immunity, plays a key role in the host’s defense against TB by promoting phagocytosis, recruiting immune cells, and generating inflammatory mediators such as C3a and C5a (3). While complement activation is essential for infection control, excessive or dysregulated activation can contribute to tissue damage and chronic inflammation, exacerbating disease progression (4). Thus, understanding the role of complement activation in TB is critical for developing strategies to modulate inflammation and enhance host defense mechanisms. Early detection and timely treatment remain vital to preventing transmission and minimizing disease severity.

Unfavorable TB treatment outcomes—including treatment failure, relapse, prolonged disease progression, and mortality—are influenced by multiple factors such as drug resistance, co-infections, immune dysfunction, and *Mtb*’s immune evasion strategies (5, 6). A comprehensive understanding of the immune pathogenesis of TB and the factors contributing to poor treatment responses is essential for improving diagnostics, optimizing therapeutic interventions, and enhancing patient adherence. Differences in immune function, co-existing conditions, and overall health can alter individual responses to TB therapy. Identifying biomarkers such as complement factors may enable personalized treatment approaches and more comprehensive patient management. Early identification of high-risk patients supports timely intervention, optimized treatment strategies, and improved clinical outcomes while helping to prevent relapse and limit TB transmission (6).

While previous studies have investigated the role of complement factors in TB, their precise contribution to pulmonary TB, particularly in patients with unfavorable treatment outcomes, remains incompletely understood. To address this gap, we conducted a nested case-control study within a cohort of pulmonary TB patients in Chennai, India, comparing individuals with favorable and unfavorable treatment outcomes. This prospective study aims to provide a detailed evaluation of the complement system’s role in TB pathogenesis and to explore potential associations between complement activity and treatment outcomes.

## Materials and methods

### Study population and sample collection

The study included 446 participants from the *Effect of Diabetes on Tuberculosis Severity* (EDOTS) study, a prospective cohort conducted in Chennai, India, between 2014 and 2019. Among these participants, 68 individuals experienced unfavorable treatment outcomes, including treatment failure, relapse, or death. Eligible participants were adults between the ages of 18 and 75 with newly diagnosed smear- and culture-positive pulmonary tuberculosis (PTB). Key exclusion criteria included prior TB treatment, drug-resistant TB, HIV infection, immunosuppressive therapy, or having received more than seven days of anti-TB treatment before enrollment. As part of the study protocol, all participants underwent HIV screening. The diagnosis of PTB was confirmed through a positive sputum culture on solid media along with a compatible chest X-ray. Anti-TB treatment was administered following the guidelines of the *Revised National Tuberculosis Control Programme* at the time. Participants were monitored monthly during the six-month treatment course and subsequently every three months for up to one year after treatment completion.

A nested case-control study was conducted, where cases were defined as individuals with adverse treatment outcomes, including 18 cases of treatment failure, 16 deaths, and 34 TB recurrences. These cases were matched in a 1:1.6 ratio with controls who had achieved a recurrence-free cure by the end of the study. Cure was defined as negative sputum cultures at both months five and six of treatment. Matching criteria included age, gender, body mass index (BMI), and diabetes status. Peripheral blood samples were collected in heparinized tubes at baseline and processed to obtain plasma for storage at -80 °C. To reduce artifactual complement activation, blood was gently inverted and processed within 2 hours of collection. Plasma was separated by centrifugation at 4 °C, aliquoted, and immediately frozen at -80 °C. Samples were subjected to minimal freeze-thaw cycles, and all handling was performed on ice whenever feasible to preserve complement integrity. Demographic and epidemiological data for this cohort have been previously reported (5).

### Measurement of complement cascade proteins and complement regulatory proteins

The levels of complement proteins, including C2, C3, C3b/iC3b, C4, C4b, C5, C5a, and mannose-binding lectin (MBL), along with complement regulatory proteins such as Factor B, Factor D, Factor H, and Factor I, were measured using bead-based multiplex complement assay kits (Luminex Corporation). The lowest detection limits for each protein were 1.4 ng/mL for C2, 0.3 ng/mL for C3, 8.2 ng/mL for C3b/iC3b, 0.6 ng/mL for C4, 1.4 ng/mL for C4b, 2.7 ng/mL for C5, 4.1 ng/mL for C5a, 0.1 ng/mL for MBL, 0.1 ng/mL for Factor B, 0.07 ng/mL for Factor D, 0.04 ng/mL for Factor H, and 0.7 ng/mL for Factor I.

### Statistical analyses

Geometric means were used to summarize central tendencies. Statistical differences between cases and controls were analyzed using the Mann–Whitney U test, while the Wilcoxon signed-rank test was used to compare complement protein levels before and during treatment. Statistical analyses were conducted using *GraphPad PRISM* (Version 9.0, GraphPad Software, Boston, MA, USA). Baseline characteristics were summarized by treatment outcome. Categorical variables were reported as frequencies (%) and compared using Pearson’s chi-squared test or Fisher’s exact test, as appropriate. To reduce skewness, biomarker values were log-transformed prior to regression modelling. Relative risks (RRs) and adjusted relative risks (aRRs) for the unfavorable outcome were estimated using generalized linear models (GLMs) with a binomial distribution and log link. Biomarkers were modelled as continuous predictors. Models were adjusted for visits, age (years), sex, body mass index (BMI, kg/m²), diabetes status, smoking, and alcohol use. To account for repeated measures within individuals, cluster-robust standard errors were applied using participant ID as the clustering variable.

### Ethics statement

The study was approved by the ethics committees of the National Institute for Research in Tuberculosis (NIRT) (012/NIRT-IEC/2015 dated. 13^th^ Feb 2015) and the Prof. M. Viswanathan Diabetes Research Center (ECR/51/INST/TN/2013/MVDRC/01 dated 23^rd^ May 2013). Written informed consent was obtained from all participants. All study procedures were conducted in accordance with institutional ethical guidelines.

## Results

### Study population

The study cohort consisted of 68 cases and 108 controls. The median age of the cases was 45 years, with an interquartile range (IQR) of 23 to 65 years, while the median age of the controls was also 45 years, with an IQR of 36 to 50 years (p = 0.526). There were no significant differences between cases and controls in terms of gender, body mass index (BMI), diabetic status, dyslipidemia, alcohol use, education level, or occupation. Additionally, no differences were observed in baseline culture smear grades or the presence of cavitary lesions. However, the control group had a significantly higher proportion of smokers, both current and former, compared to the case group (p = 0.0019) [34]. (**Table 1**).

**Table 1:** Patients characteristics for individuals with TB treatment outcome.

	Control (Favorable) (n=108)	Cases (Unfavorable) (n=68)	Sig.
Age in Years	45 (36 - 50)	45 (23 - 65)	0.526
Gender			
Female	21 (19.4)	8 (11.8)	0.181
Male	87 (80.6)	60 (88.2)	
BMI Classification			
<18.5 kg/m^2^	63 (58.3)	47 (69.1)	0.150
≥18.5 kg/m^2^	45 (41.7)	21 (30.9)	
Diabetes Status			
Non-Diabetes	45 (41.7)	26 (38.2)	0.651
Diabetes	63 (58.3)	42 (61.8)	
Cough			
Absence	2 (1.9)	1 (1.5)	0.849
Presence	106 (98.1)	67 (98.5)	
Dyslipidaemia			
Absence	108 (100)	68 (100)	NA
Presence	0 (0)	0 (0)	
Smoking			
Never	61 (56.5)	26 (38.2)	**0.019**
Past	23 (21.3)	14 (20.6)	
Current	24 (22.2)	28 (41.2)	
Alcohol			
Never	38 (35.2)	16 (23.5)	0.193
Past	22 (20.4)	13 (19.1)	
Current	48 (44.4)	39 (57.4)	
Cavity			
Absence	66 (61.1)	40 (58.8)	0.662
Presence	31 (28.7)	18 (26.5)	
Not Known	11 (10.2)	10 (14.7)	
Smear			
1+	73 (67.6)	36 (52.9)	**0.099**
2+	32 (29.6)	27 (39.7)	
3+	3 (2.8)	5 (7.4)	
Culture			
1+	45 (41.7)	25 (36.8)	0.429
2+	21 (19.4)	10 (14.7)	
3+	42 (38.9)	33 (48.5)	

Values were presented as n(%) and median (first - third quartile).

Fisher Exact and Mann-Whitney test were used to check the significance. p value <0.05 is significant and p value >0.05 is not significant.

Bold text denotes statistically significant *p* values.

### Altered complement activation in pulmonary TB patients with unfavorable treatment outcomes

**Figure 1:**
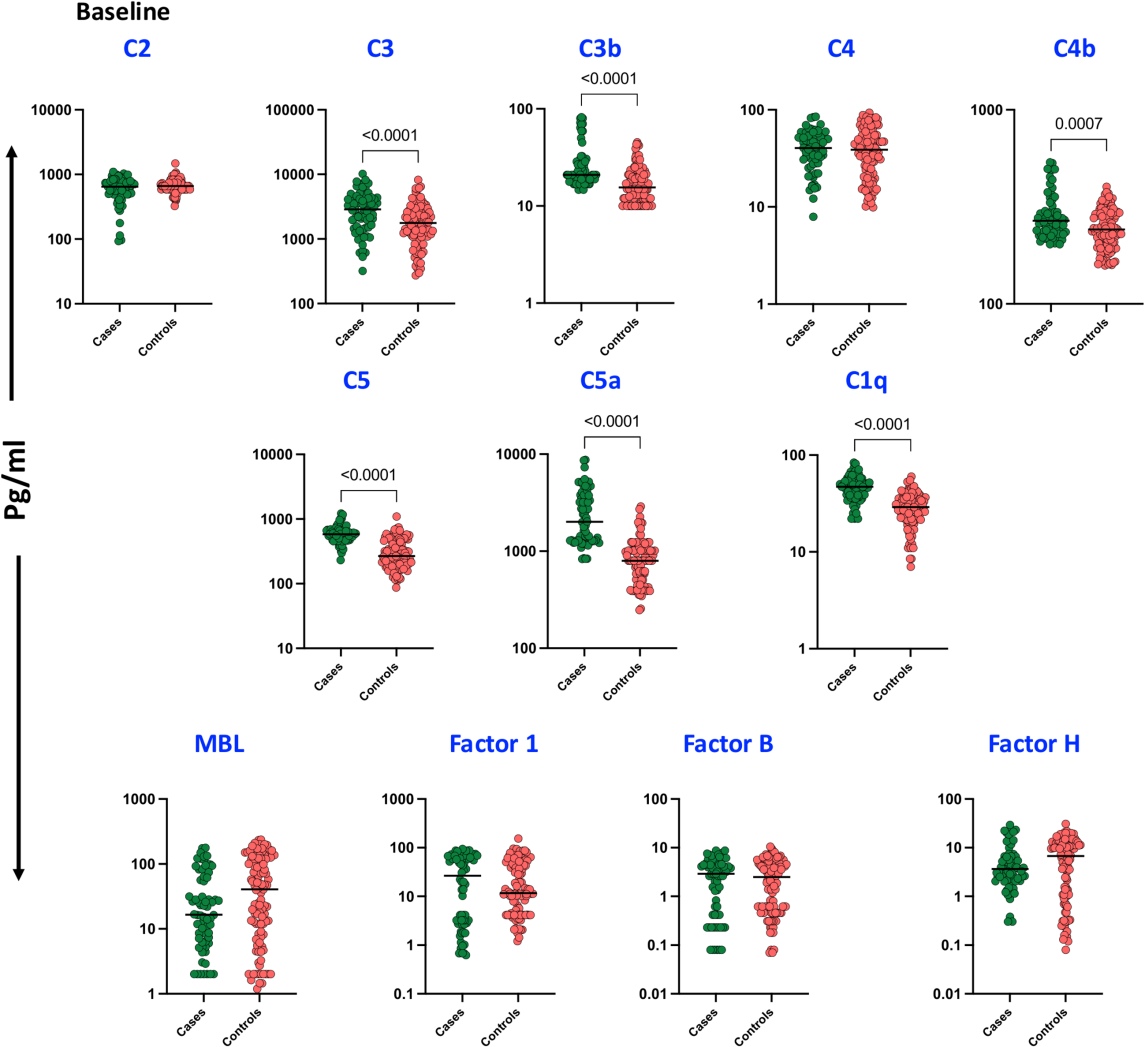
Complement proteins and Complement Regulatory Proteins in TB patients with unfavorable TB treatment outcome and treatment cured controls at Baseline: Plasma levels of Complement proteins and Complement Regulatory Proteins were measured in cases (n = 68) and controls (n = 108) These data were represented in a box plot where each dot represents every single participant in its group. The median line was presented at the middle of the graphs. Mann–Whitney test was used to calculate the p-values of the unrelated groups.

To assess the baseline levels of plasma complement proteins, including C2, C3, C3b/iC3b, C4, C4b, C5, C5a, mannose-binding lectin (MBL), and complement regulatory proteins such as Factor B, Factor D, Factor H, and Factor I, a comparative analysis was conducted between cases and controls. As shown in **Figure 1**, baseline plasma levels of C3 (p < 0.0001), C3b (p < 0.0001), C4b (p = 0.0007), C5 (p < 0.0001), C5a (p < 0.0001), and C1q (p < 0.0001) were significantly elevated in cases compared to controls. However, no significant differences were observed in the plasma levels of C2, C4, MBL, Factor I, Factor B, and Factor H.

**Figure 2:**
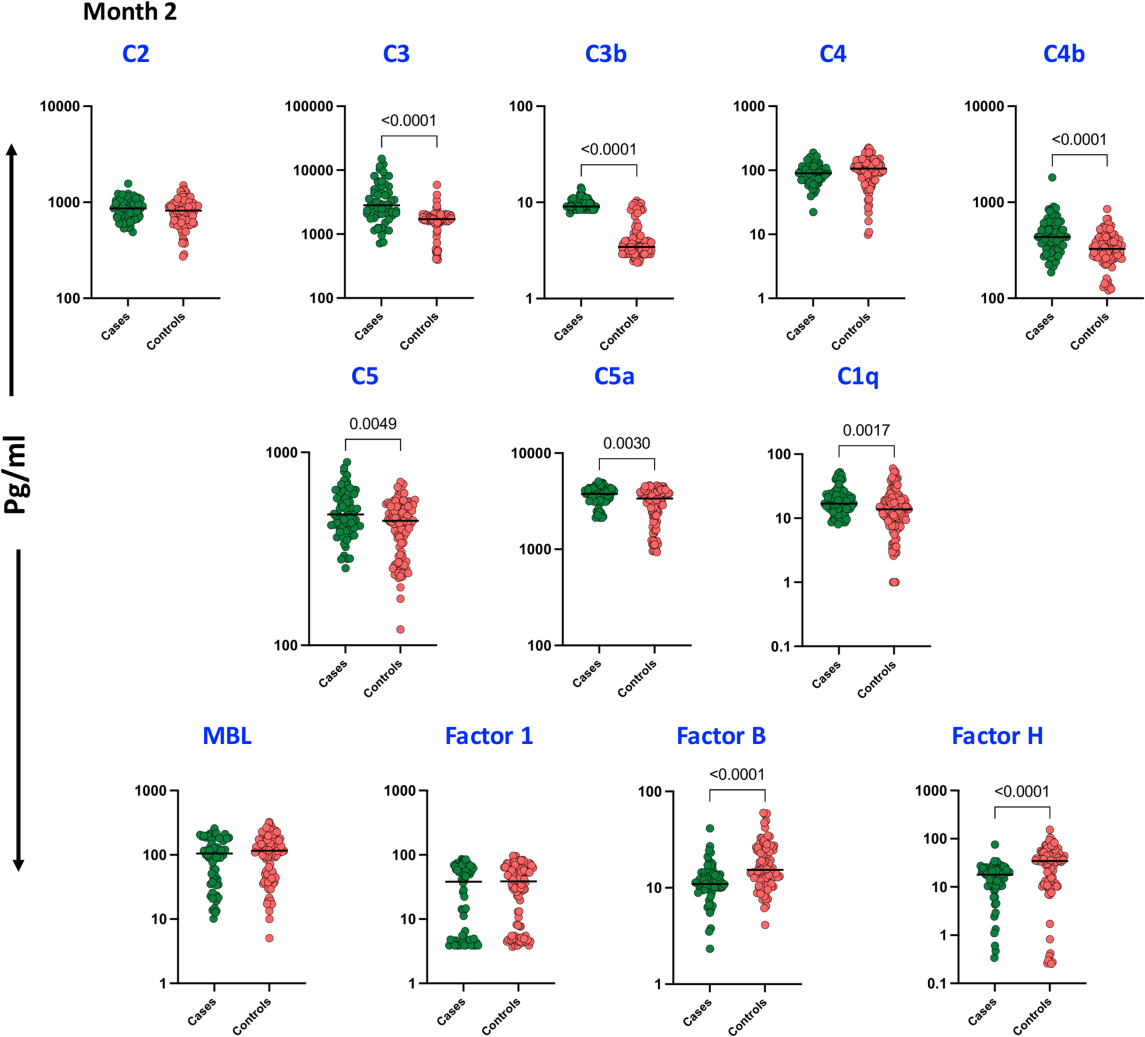
Complement proteins and Complement Regulatory Proteins in TB patients with unfavorable TB treatment outcome and treatment cured controls at Month 2: Plasma levels of Complement proteins and Complement Regulatory Proteins were measured in cases (n = 68) and controls (n = 108) These data were represented in a box plot where each dot represents every single participant in its group. The median line was presented at the middle of the graph. Mann–Whitney test was used to calculate the p-values of the unrelated groups.

A similar trend was observed during the second month of anti-TB treatment, as illustrated in **Figure 2**. Plasma levels of C3 (p < 0.0001), C3b (p < 0.0001), C4b (p = 0.0007), C5 (p = 0.0005), C5a (p = 0.0030), and C1q (p = 0.0017) remained significantly elevated in cases compared to controls. However, levels of the complement regulatory proteins Factor B (p < 0.0001) and Factor H (p < 0.0001) were significantly reduced in cases, suggesting altered inflammatory responses during the course of anti-TB treatment. No significant differences were observed in the plasma levels of C2, C4, MBL, and Factor I.

### Association of the biomarker with the unfavorable treatment outcomes

To define the plasma complement factors that correlate with unfavorable treatment outcomes, we performed univariate and multivariate conditional regression analyses, with the latter correcting for age, gender, BMI, diabetic status, smoking, alcohol use, presence of cavity, smear and culture status, and occupation (**Table 2**). Univariate analysis showed that C4b (odds ratio [OR],2.34; 95% confidence interval [CI], 1.82–3.01; P = <0.001), C5 (OR, 2.85; 95% CI, 2.32–3.50; P <.001), C5a (OR, 1.63; 95% CI, 1.37–1.94; P <.001), C1q (OR, 2.17; 95% CI, 1.74–2.70; P <.001), C3 (OR, 2.10; 95% CI, 1.77–2.51; P <.001), C3b (OR, 1.41; 95% CI, 1.29–1.54; P <.001) were associated with increased rates of unfavorable treatment outcomes. Multivariate analysis showed that C4b (adjusted OR [aOR], 4.02; 95% CI, 2.45–6.61; P <.001), C5 (aOR, 3.31; 95% CI, 2.37–4.61; P <.001), C5a (aOR, 1.64; 95% CI, 1.36–1.98; P <.001), C1q (aOR, 2.43; 95% CI, 1.85–3.19; P <.001), C3 (aOR, 2.18; 95% CI, 1.65–2.89; P <.001), C3b (aOR, 2.29; 95% CI, 2.29–2.29; P <.001) were still associated with significantly increased risk of unfavorable treatment outcomes.

**Table 2:** Association of the biomarker with the unfavorable treatment outcomes.

Marker	Univariate model		Multivariate model	
	RR (95% CI)	p-Value	aRR^a^ (95% CI)	P-Value
C2	0.93 (0.78, 1.11)	0.426	0.92 (0.75, 1.11)	0.371
C4b	2.34 (1.82, 3.01)	**<0.001**	4.02 (2.45, 6.61)	**<0.001**
C5	2.85 (2.32, 3.50)	**<0.001**	3.31 (2.37, 4.61)	**<0.001**
MBL	0.92 (0.85, 0.99)	0.048	0.91 (0.82, 1.01)	0.076
FactorI	0.94 (0.83, 1.06)	0.301	0.93 (0.82, 1.06)	0.288
C5a	1.63 (1.37, 1.94)	**<0.001**	1.64 (1.36, 1.98)	**<0.001**
C1q	2.17 (1.74, 2.70)	**<0.001**	2.43 (1.85, 3.19)	**<0.001**
C3	2.10 (1.77, 2.51)	**<0.001**	2.18 (1.65, 2.89)	**<0.001**
C3b	1.41 (1.29, 1.54)	**<0.001**	2.29 (2.29, 2.29)	**<0.001**
C4	0.98 (0.88, 1.10)	0.756	0.99 (0.85, 1.16)	0.905
FactorB	0.97 (0.92, 1.03)	0.343	0.89 (0.78, 1.01)	0.067
FactorH	0.96 (0.91, 1.01)	0.076	0.95 (0.89, 1.02)	0.145

CI, confidence interval; RR, Risk Ratio.

a Risk Ratio was estimated after adjusted for age in years, gender, body mass index, diabetes status, smoking status, and alcohol status.

Bold text denotes statistically significant *p* values.

### Plasma complement proteins are also biomarkers for individual treatment outcomes in PTB

**Figure 3:**
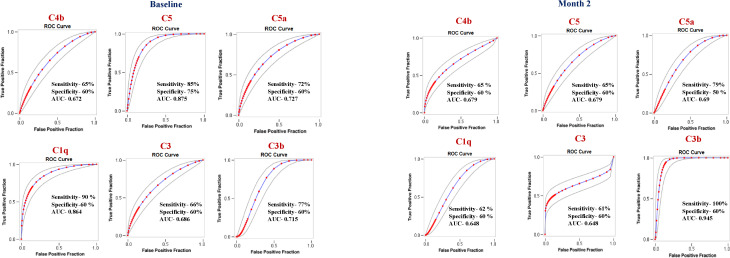
Plasma complement factors are biomarkers of individual treatment outcomes in active tuberculosis (TB) disease: Receiver operating characteristic (ROC) analysis was conducted to evaluate the sensitivity, specificity, and area under the curve (AUC) of complement factors measured at baseline and at month 2, assessing their ability to discriminate between TB recurrence and recurrence-free cure. Only complement factors demonstrating statistically significant discriminatory performance (P < 0.05) are presented. AUC, area under the curve.

To determine if we could derive a signature of plasma complement factors that could be used as a biomarker for individual treatment outcomes (TB recurrence, TB relapse, and mortality), we performed ROC analysis of complement factors. As shown in **Figure 3**, baseline ROC analysis of C4b (AUC = 0.672), C5 (AUC = 0.875), C5a (AUC = 0.727), C1q (AUC = 0.864), C3 (AUC = 0.686) and C3b (AUC = 0.715) exhibited increased sensitivity and specificity in differentiating TB recurrence vs recurrence-free cure. Similarly, as shown in **Figure 3** for the month 2, ROC analysis of C4b (AUC = 0.679), C5 (AUC = 0.679), C5a (AUC = 0.690), C1q (AUC = 0.648), C3 (AUC = 0.648) and C3b (AUC = 0.945) exhibited increased sensitivity and specificity in differentiating TB.

### Altered complement components before and after intensive phase treatment in cases and controls

**Figure 4:**
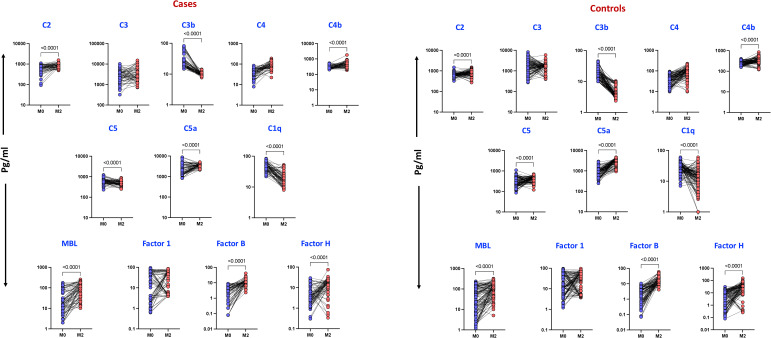
Altered Complement proteins and Complement Regulatory Proteins levels during 2^nd^ month of anti-TB treatment in cases and Controls: The plasma levels of complement components were measured in Controls at baseline (pre) and during 2 months following treatment (post). The data are presented as line graphs with each line representing a single individual. P values were calculated using the Wilcoxon signed rank test.

To investigate changes in complement component levels before and after intensive phase anti-TB treatment (ATT) at month 2, we compared cases and controls. As shown in **Figure 4**, cases exhibited significant elevations in C2 (p < 0.0001), C4b (p < 0.0001), MBL (p < 0.0001), Factor B (p < 0.0001), and Factor H (p < 0.0001), whereas C3b (p < 0.0001), C5 (p < 0.0001), C5a (p = 0.0013), and C1q (p < 0.0001) were significantly diminished. In the control group, as shown in **Figure 4**, C2 (p < 0.0001), C4b (p < 0.0001), C5a (p < 0.0001), MBL (p < 0.0001), Factor B (p < 0.0001), and Factor H (p < 0.0001) were significantly elevated, whereas C3b (p < 0.0001), C5 (p < 0.0001), and C1q (p < 0.0001) were significantly diminished. These results highlight distinct alterations in complement activation, suggesting a role for the complement system in the host immune response to pulmonary tuberculosis.

## Discussion

The immune response to tuberculosis (TB) is complex, with the complement system playing a crucial role in both pathogen defense and immune regulation (6). Recent studies have highlighted complement components such as C1q as potential biomarkers for diagnosing pulmonary and extrapulmonary TB, as well as for detecting active disease. Complement activation influences immune cells, including dendritic cells, macrophages, and T cells, through key fragments like C3a and C5a (7, 8). While complement activation is essential for immune defense—facilitating immune cell recruitment, opsonization, and pathogen clearance—its dysregulation can lead to excessive inflammation and tissue damage, potentially contributing to disease persistence (4, 8).

Elevated levels of complement proteins, including C3, C4, C5, and C5a, are commonly observed in active TB, reflecting ongoing immune activation (8). However, while this response aids in infection control, it can also drive granuloma formation and chronic inflammation. As demonstrated in our study, significant alterations in complement and regulatory proteins were observed before and after anti-TB treatment (ATT), suggesting that a balanced inflammatory response is critical for optimal immune pathology. Overactivation or poor regulation of the complement system can result in excessive tissue damage, fibrosis, and chronic inflammation, which are linked to unfavorable TB outcomes, including cavitary disease (3, 9). Additionally, M. tuberculosis has evolved mechanisms to evade complement-mediated clearance, further complicating host-pathogen interactions (4).

Mannose-binding lectin (MBL) plays a key role in recognizing M. tuberculosis, triggering complement activation via the lectin pathway. This leads to C3b deposition, promoting opsonization and bacterial uptake by phagocytes (10). Factor I regulates this process by inactivating C3b and C4b, preventing excessive complement activation and limiting tissue damage (4, 11). The alternative pathway, continuously active at low levels, amplifies the complement cascade through Factor B and Factor H (12, 13). Despite their regulatory roles, our study found no significant differences in MBL, Factor B, Factor H, or Factor I levels between cases and controls, suggesting these proteins are not major contributors to disease progression.

Unfavorable TB treatment outcomes, including treatment failure, relapse, and drug resistance, are often associated with dysregulated immune responses (14, 15). Excessive complement activation may exacerbate inflammation and hinder bacterial clearance, contributing to poor outcomes [30]. Identifying complement proteins as inflammatory biomarkers could help refine TB treatment strategies and improve patient prognosis.

The altered complement profiles in pulmonary TB patients with unfavorable outcomes suggest dysregulation of both the classical and alternative pathways. Elevated C1q, C4b, and C3b levels indicate sustained classical pathway activation, potentially driving chronic inflammation and tissue damage. Concurrently, reduced Factor B and Factor H levels suggest impaired regulation of the alternative pathway, leading to excessive complement activation. This imbalance may contribute to poor immune control and adverse treatment outcomes. Modulating complement activation or enhancing regulatory mechanisms could offer a promising adjunctive strategy in TB management (4, 8).

In parallel, the reduced levels of Factor B and Factor H—key components and regulators of the alternative pathway—indicate impaired regulation of complement amplification. Factor B is essential for the formation of the C3 convertase in the alternative pathway, while Factor H is a major negative regulator that protects host tissues from excessive complement activity (16, 17). Their depletion may lead to uncontrolled complement activation, promoting inflammatory damage rather than effective mycobacterial clearance. These findings suggest that imbalance between activation (particularly via the classical pathway) and regulation (via the alternative pathway) may underlie the exaggerated immune responses observed in patients with poor treatment outcomes. Targeting specific arms of the complement system, such as modulating classical pathway activation or enhancing alternative pathway regulation, could represent a novel adjunctive approach to improving TB treatment responses and reducing immunopathology. An early onset of complement activation, especially through the classical pathway as indicated by our results, may be associated with poorer treatment outcomes. Additionally, it is important to consider the factors that contribute to this activation. Our findings show that several complement components are significantly associated with unfavorable TB treatment outcomes, indicating their potential as prognostic as candidate biomarkers, but are not yet validated for biological relevance or clinical use. However, we acknowledge that functional studies and validation in independent cohorts are required to clarify the biological role of these complement components and to establish their predictive or clinical utility. Given the observational nature of this study, no causal or disease-driving mechanisms can be inferred. Future studies integrating complement markers with conventional inflammatory and clinical indicators are needed to determine whether complement measurements provide independent or added predictive value. This represents a limitation of the current study. Additionally, we did not perform functional assays to confirm pathway-specific activation, such as distinguishing between classical and alternative pathways. Furthermore, heparin plasma was used instead of EDTA, which may allow some ex vivo complement activation despite careful sample handling.

This study showed that complement activation patterns strongly align with the objective of identifying immune markers linked to poor TB treatment outcomes. Patients with unfavorable outcomes displayed elevated baseline levels of key complement proteins (C3, C3b, C4b, C5, C5a, C1q) and reduced regulatory factors, indicating early immune dysregulation. These abnormalities persisted during treatment, unlike in controls who showed improved regulation. Regression analyses confirmed these markers as independent predictors of treatment failure, relapse, or death. Differences in complement dynamics between baseline and month two further highlighted impaired immune resolution in cases, supporting their potential as prognostic biomarkers. This work as an independent, hypothesis-generating study focused specifically on quantifying the complement components, at the baseline or before initiating the Treatment and their association with treatment outcomes. Future studies could explore combined biomarker models, but that such analyses are beyond the scope of the present work and would require dedicated validation in independent cohorts.

These findings indicate persistent complement activation in these patients, suggesting a dysregulated or prolonged inflammatory response. Additionally, we observed consistently reduced levels of regulatory proteins, particularly Factor B and Factor H, in cases by month two of treatment, pointing to impaired complement regulation. This imbalance—characterized by excessive activation and insufficient regulation may contribute to heightened inflammation and tissue damage, rather than effective pathogen clearance. These results underscore the potential of the complement system as a therapeutic target in TB. Modulating complement activity, either by dampening overactivation or enhancing regulatory mechanisms, could serve as a valuable adjunct to current TB treatment strategies. Such interventions may help control inflammation, minimize tissue injury, and promote more efficient bacterial clearance. Further investigation is warranted to better understand the mechanistic links between complement activation, TB disease progression, and treatment outcomes.

## Conclusions

In sum, our study has identified or quantified the baseline complement markers and evaluated the association between their circulating levels and the treatment outcomes. Complement dysregulation can be associated with unfavorable treatment outcomes in pulmonary tuberculosis. Elevated levels of classical pathway components (C3, C3b, C4b, C5, C5a, and C1q), at the baseline or before the start of the treatment, may serve as potential prognostic biomarkers for identifying patients at increased risk of poor outcomes. These findings indicate association rather than causality, and further experimental and longitudinal mechanistic studies are needed to validate their clinical utility and clarify their biological role.

## Data Availability

The original contributions presented in the study are included in the article/supplementary material. Further inquiries can be directed to the corresponding author/s.
